# Prise en charge péri-opératoire de l’atrésie de l’œsophage: petits pas non négligeables à Madagascar

**DOI:** 10.11604/pamj.2017.27.9.10817

**Published:** 2017-05-05

**Authors:** Harifetra Mamy Richard Randriamizao, Aurélia Rakotondrainibe, Nadia Marie Philibertine Rahanitriniaina, Andriambelo Tovohery Rajaonera, Mamy Lalatiana Andriamanarivo

**Affiliations:** 1 Service de Réanimation Chirurgicale, CHU-HJRA BP 4150, Antananarivo, Faculté de Médecine d’Antananarivo, Université d’Antananarivo, Madagascar; 2 Service de Chirurgie Viscérale Pédiatrique, CHU-HJRA BP 4150, Antananarivo, Faculté de Médecine d’Antananarivo, Université d’Antananarivo, Madagascar

**Keywords:** Atrésie de l´œsophage, Madagascar, mortalité, précarité, survie, Esophageal atresia, Madagascar, mortality, precariousness, survival

## Abstract

La prise en charge de l'atrésie de l'œsophage est encore limitée par la précarité des plateaux techniques à Madagascar. Les cas décrits dans ce travail ont pour objectif de relater nos possibilités thérapeutiques et de décrire les progrès à réaliser pour optimiser le traitement de cette pathologie congénitale. Nous avons recueilli tous les dossiers ayant pour motif d’entrée au service de Réanimation Chirurgicale du CHU JRA, Antananarivo, une atrésie de l’œsophage. Nous en avons retenu les tous premiers cas qui ont survécu sur une période de 42 mois entre janvier 2011 et juin 2014. Parmi 17 admissions pour atrésie de l’œsophage, trois nouveau-nés à terme, admis successivement en Réanimation Chirurgicale, présentant un type III d'atrésie; premiers patients, à Madagascar, ayant survécu au décours de leur intervention. Une seule patiente avait présenté des malformations associées. Ces trois bébés ont été extubés précocement au bloc opératoire, sous oxygénothérapie jusqu'à une ventilation spontanée efficace. Des séances de kinésithérapie postopératoire permettaient d'améliorer l'état respiratoire des nouveau-nés. La mortalité globale de cette pathologie en 42 mois a été de 76,5%. Malgré ces premiers succès, des progrès restent à entreprendre dans le traitement de cette anomalie congénitale ; de son diagnostic jusqu'à la période postopératoire. L'amélioration du plateau technique, surtout ventilatoire et du support nutritionnel permettrait d'avoir des résultats plus probants, tout comme dans les pays où des progrès ont été réalisés sur le plan de la réanimation.

## Abstract (en)

The management of esophageal atresia is still limited due to the precariousness of technical equipments in Madagascar. Our case study aims to highlight possible therapeutic options and to describe the progresses to be made so as to optimize treatment of this congenital pathology. We collected the medical records of all patients hospitalized for esophageal atresia in the Department of Surgical Reanimation at the University Hospital-JRA Antananarivo. The first patients admitted who survived for a period of 42 months (between January 2011 and June 2014) were included in the study. Among the 17 patients admitted with esophageal atresia, three full-term newborns with type III esophageal atresia, successively admitted in Surgical Reanimation, were the first patients in Madagascar to survive after surgery. Only one patient had associated malformations. These three babies were extubated early in the operating room; they underwent oxygen therapy until normal spontaneous breathing. New-born babies underwent post-operatory physical therapy allowing to improve their respiratory status. Overall mortality rate from this pathology was 76.5% in 42 months. Despite these initial success, progresses still remain to be done in the treatment of this congenital anomaly, from diagnosis up to postoperative period. The improvement of technical equipments, especially ventilation and nutritional support equipments would allow more meaningful results, like in the countries where progresses has been made in the field of reanimation.

## Introduction

La prise en charge de l'atrésie de l'œsophage représente un grand défi anesthésique per et postopératoire [1–3]. Dans les pays développés, la mortalité autrefois élevée a considérablement baissé, grâce aux progrès de la réanimation néonatale [4]. Cependant, dans les pays en voie de développement cette mortalité reste encore importante [5–7]. A Madagascar, les plateaux techniques pour la prise en charge péri-opératoire des nouveau-nés sont très limités. Aucun chiffre n'a encore été édicté concernant la prise en charge péri-opératoire de l'atrésie de l'œsophage, notamment du point de vue survie et prise en charge en réanimation. Notre objectif est de présenter les premiers bébés ayant survécu à la cure chirurgicale de cette pathologie, à Madagascar.

## Méthodes

Nous avons analysé les dossiers des bébés admis pour atrésie de l’œsophage entre janvier 2011 et juin 2014, au service de Réanimation Chirurgicale du CHU JRA, Antananarivo, Madagascar. Nous avons retenu ceux qui ont survécu, pour mettre en exergue les moyens thérapeutiques utilisés. Nous en rapportons les cas observés des trois premiers survivants, successifs, au cours de cette période. Cette présentation de cas vise à décrire les moyens que nous avons utilisés, mais également les progrès qui restent à entreprendre pour optimiser la prise en charge de cette anomalie dans un centre hospitalier d'Antananarivo (Madagascar).

## Résultats

Durant ces 42 mois, 17 nouveau-nés ont été admis au service de réanimation chirurgicale. Leur admission médiane de 3 [1–9] jours après la naissance, était directe. La mortalité globale était de 76,5%. A partir de 2012, seuls trois bébés nés à terme, avec une atrésie de type III, ont survécu après œsophagoplastie. Par manque de suppléance respiratoire néonatale postopératoire, nous avons opté pour une extubation au bloc opératoire, dès le réveil post-anesthésique. Le sevrage en oxygène s'est fait lorsque la saturation en oxygène à 21% était supérieure à 92%, sans détresse respiratoire. La reprise de l'alimentation a été constituée par du lait maternel, via une sonde gastrique trans-anastomotique, associée à un apport intra-veineux d'acides aminés. Outre les mesures de réanimation, des séances de kinésithérapie journalières, voire biquotidiennes, ont été instaurées. Le premier nouveau-né, un garçon, pesant 2550 g, était admis au 2^ème^ jour de sa naissance. L'intervention a été réalisée au 4^ème^ jour. L'entretien de l'anesthésie s'est fait sous halothane, sous ventilation assistée au ballon avec une pression expiratoire positive à 5cmH2O. L'enfant était extubé 30 minutes après l’intervention. Le gavage était programmé au premier jour postopératoire (J1). Le séjour en réanimation a été de 16 jours. Avec un recul de 20 mois, le développement de l'enfant s’est fait normalement. Le 2^ème^ nouveau-né, 2600g, de sexe masculin était admis à la 6^ème^ heure de sa naissance. L'extubation a été réalisée 60 minutes après la fin de l'acte chirurgical, la reprise alimentaire programmée à J1. Le patient a été transféré à J7. Avec un recul de 10 mois, l'enfant se développait sans problème, jusqu'à sa perte de vue. Le troisième nouveau-né, une fille de 2300g ; opérée sous anesthésie générale intraveineuse précédée de crash induction, à J2 de vie, extubée 120 minutes après la fin de l'intervention. La réanimation postopératoire a duré 19 jours, avant son transfert. Une crâniosténose, une hépatomégalie et en postopératoire des communications inter-cavitaires avec transposition des gros vaisseaux ont été décelés. Une infection pulmonaire installée un jour après l'intervention s'est amendée après antibiothérapie adaptée et séances de kinésithérapie biquotidiennes.

## Discussion

Nous rapportons les premiers cas d’atrésie de l’œsophage ayant survécu à une cure chirurgicale, à Madagascar. L'atrésie de l'œsophage touche près de 1 sur 2500 à 4000 naissances vivantes, depuis 30-40 ans [4]. L'évolution de sa prise en charge s'est nettement améliorée, dans les pays développés grâce à un diagnostic très précoce, voire prénatal, à l'amélioration de la réanimation néonatale; ce qui n'est pas le cas dans les pays en voie de développement [4, 9] comme Madagascar. Nous avons retrouvé un taux de mortalité de 76,5% en 3 ans et 6 mois et un retard de prise en charge (admission à trois jours de la naissance), mais également un cadrage incomplet (pas de bilan malformatif préopératoire). Les trois premiers nouveau-nés ayant survécu durant la période d'étude, démontrent la précarité des plateaux techniques disponibles, outre les possibilités de prise en charge, adaptées à notre contexte. Cette situation n'est pas propre à notre milieu. Dans des pays africains, le taux de mortalité allait de 39,2% au Mahgreb [6] à 76 à 90% en Afrique sub-saharienne [7, 8]. En Afghanistan, ayant un système de santé très précaire, trois succès de cure d’atrésie de l'œsophage de type III ont été relatés depuis 2005 [5]. Les causes de ce fort taux de mortalité pourraient être expliquées par un dépistage non systématique de cette pathologie avec l'installation d'une pneumopathie d'inhalation, mais aussi à un plateau technique et une réanimation néonatale précaires [5–9]. Pourtant dans les pays plus avancés, une diminution de la mortalité a pu être constatée grâce à l'amélioration du dépistage de cette pathologie et des anomalies associées, plus particulièrement les cardiopathies. En Inde, dans l'étude de Sharma et al [10], elle est passée globalement de 91,5% à 41% en 25 ans. Au Moyen-Orient, sur des périodes de 10 à 15 ans, la mortalité globale se situait entre 19,5% et 56% [9, 11, 12]. Elle augmente lorsque une malformation était associée, mais également du fait d'une intervention tardive, du faible poids de naissance et/ou de prématurité ainsi que des complications postopératoires [1, 9, 12]. En Asie, les avancées de la prise en charge de l'atrésie de l'œsophage, notamment au niveau de la réanimation néonatale (ventilation, support nutritionnel), ont fait que la mortalité est entre 12,5% et 23% sur des périodes de 8 à 15 ans [3, 13–15]. Les causes de décès retrouvées restaient les malformations cardiaques surtout, ainsi que les pneumopathies sévères [13–15]. Dans les pays développés européens et en Australie, la mortalité a nettement diminué au fil des années, allant de 28,2%-66% dans les années 60; à 33% dans les années 70; à 14% dans les années 80 voire entre 1 et 10% dans les années 90 [2, 16, 17]. Bien que l'amélioration du support ventilatoire postopératoire ait amélioré le taux de survie; les causes de décès se révélaient toujours être les cardiopathies congénitales majeures [9, 16, 17]. Ce support ventilatoire chez les nouveau-nés n’étant pas disponible à Antananarivo (Madagascar), l’extubation au bloc et l’optimisation ventilatoire en postopératoire, par des séances de kinésithérapie respiratoire entre autre, ont été bénéfiques pour nos patients. De plus, la nutrition artificielle (parentérale notamment) disponible dans nos officines étant inadaptée aux nouveau-nés, l’apport de lait maternel par la sonde trans-anastomotique a permis l’apport nutritionnel dès le premier jour postopératoire. Cependant, cette pathologie entre souvent dans le cadre de syndrome polymalformatif; aussi, un bilan malformatif complet, surtout cardiaque, s'avère nécessaire, ce qui permettrait de classifier les patients pour en déterminer la survie probable [2].

## Conclusion

L’atrésie de l’œsophage n’est pas une fatalité. La prise en charge s´est nettement améliorée en deux à trois décennies dans les pays développés et est en train de se réaliser dans les pays asiatiques et ceux du Moyen-Orient. Dans les pays pauvres, il serait primordial de limiter les retards de diagnostic et d´intervention, d´établir un bilan malformatif complet et de mieux pourvoir les plateaux techniques, notamment en réanimation néonatale, pour de meilleurs résultats. A Madagascar, des efforts restent encore à entreprendre pour diminuer le taux de mortalité lié à la cure de l´atrésie de l´œsophage. Allier connaissances et adaptation aux moyens techniques permet d’avoir des résultats probants. L’extubation précoce et l’optimisation respiratoire par des séances de kinésithérapie respiratoire ont permis à ces bébés de survivre à leur intervention; un petit pas non négligeable dans l’avancée péri-anesthésique de cette malformation congénitale à Madagascar.

### Etat des connaissances actuelles sur le sujet

- Les chances de survie dans la prise en charge de l’atrésie de l’œsophage sont disparates selon le niveau économique d’un pays: elles sont moins importantes dans les pays en voie de développement;
- Le succès du traitement de l’atrésie de l’œsophage est lié aux moyens techniques en réanimation, notamment du point de vue respiratoire, une réanimation respiratoire, avec assistance ventilatoire est nécessaire;
- Un support nutritionnel optimal est essentiel dans la prise en charge des atrésies de l’œsophage en réanimation: il est adapté au nouveau-né et est constitué entre autre d’une alimentation parentérale.

### Contribution de notre étude à la connaissance

- Cet article relate les premiers nouveau-nés ayant survécu à une cure de l’atrésie de l’œsophage à Madagascar, malgré les lacunes matérielles en termes de réanimation néonatale, surtout du point de vue assistance ventilatoire;
- La prise en charge postopératoire de l’atrésie de l’œsophage a été marquée par une extubation précoce, dès le réveil de l’enfant, au bloc opératoire: nous n’avons pas eu recours à une assistance ventilatoire mécanique postopératoire, l’optimisation respiratoire a été assurée par des séances de kinésithérapie respiratoire jusqu’à biquotidiennes;
- Devant le manque de support nutritionnel adapté au nouveau-né, à Madagascar, la prise en charge nutritionnelle était basée sur l’apport de lait maternel par gavage, par une sonde placée en trans-anastomotique par les chirurgiens. La reprise de l’alimentation a été très précoce, dès le premier jour postopératoire, par du lait maternel à travers la sonde.

## Conflits d’intérêts

Les auteurs ne déclarent aucun conflit d'intérêt.
